# Clinical response to adding pyrotinib to pembrolizumab and lenvatinib for HER2-positive advanced intrahepatic cholangiocarcinoma: a case report

**DOI:** 10.1186/s12957-023-02983-1

**Published:** 2023-03-27

**Authors:** Jun-Wei Zhang, Xiaobo Yang, Boju Pan, Yiyao Xu, Xin Lu, Hai-tao Zhao

**Affiliations:** 1 Department of Liver Surgery, State Key Laboratory of Complex Severe and Rare Diseases, Peking Union Medical College Hospital, Chinese Academy of Medical Science and Peking Union Medical College, Beijing, China; 2 Department of Pathology, Peking Union Medical College Hospital, Chinese Academy of Medical Science and Peking Union Medical College, Beijing, China

**Keywords:** Clinical response, Intrahepatic cholangiocarcinoma, Immunotherapy, Targeted therapy, HER2, Case report

## Abstract

**Background:**

Intrahepatic cholangiocarcinoma (ICC) is a highly lethal hepatobiliary cancer, and very few patients can undergo surgery. The prognosis of advanced ICC is poor, especially in patients who progress after first-line chemotherapy, with a median overall survival of less than 10 months.

**Case presentation:**

A 64-year-old male was diagnosed with advanced intrahepatic cholangiocarcinoma with ERBB2 (HER2) 3 + amplification determined by tissue-based testing and confirmed by next-generation sequencing. The patient was treated with pyrotinib added to pembrolizumab and lenvatinib after progressing with pyrotinib and tegafur and responded very well with regression of the tumor on imaging as well as normalization of tumor marker levels without serious adverse events. PET-CT after 6 months of treatment showed a partial response. The progression-free survival with second-line treatment was 17 months. For the third line of therapy, lenvatinib and pembrolizumab were used in combination with bevacizumab. Currently, he has had stable disease for approximately 6 months during third-line treatment.

**Conclusion:**

Adding pyrotinib to pembrolizumab and lenvatinib may represent a promising strategy for advanced ICC patients who have high levels of HER2.

## Introduction

Intrahepatic cholangiocarcinoma (ICC), a subtype of cholangiocarcinoma, is the second most common primary liver malignancy, with increasing global incidence. Because of the frequent absence of symptoms in the early stage of the disease, only 22% of patients are able to undergo surgery [1]. In cases of advanced biliary tract cancer (BTC), the current standard of care is systemic chemotherapy. However, chemotherapy is usually complicated with more adverse events, and for patients with metastatic BTC who experience progression even with chemotherapy, there is a lack of efficacious protocols for second-line and higher-line treatment [2].

Anti-HER2 molecular-targeted agents have been established for treating cancers with HER2 gene amplification and have been mostly used in breast cancer [3]. A combination of two HER2-targeted therapies has also been demonstrated to have clinical efficacy in advanced HER2-positive metastatic gastric and colorectal cancers [4, 5]. Recently, pertuzumab and trastuzumab were used for HER2-positive metastatic BTC (*n* = 39), with an objective response rate of 23% and no treatment-related serious adverse events or deaths [6]. The progression-free survival of ICC was 2.6 months, which was worse than that of other cholangiocarcinomas in the study [6]. As a second-line therapy, HER2-targeted therapy may need to be combined with other therapies for ICC. PD-L1 blockades have been reported to enhance the efficacy of anti-ERBB therapy [7]. We assumed that HER2 amplification limited the response of lenvatinib for BTC. In our case, combining the use of HER2-targeted therapy with lenvatinib for refractory BTC achieved an excellent response, which may prove our assumption.

## Case report

**Fig. 1:**
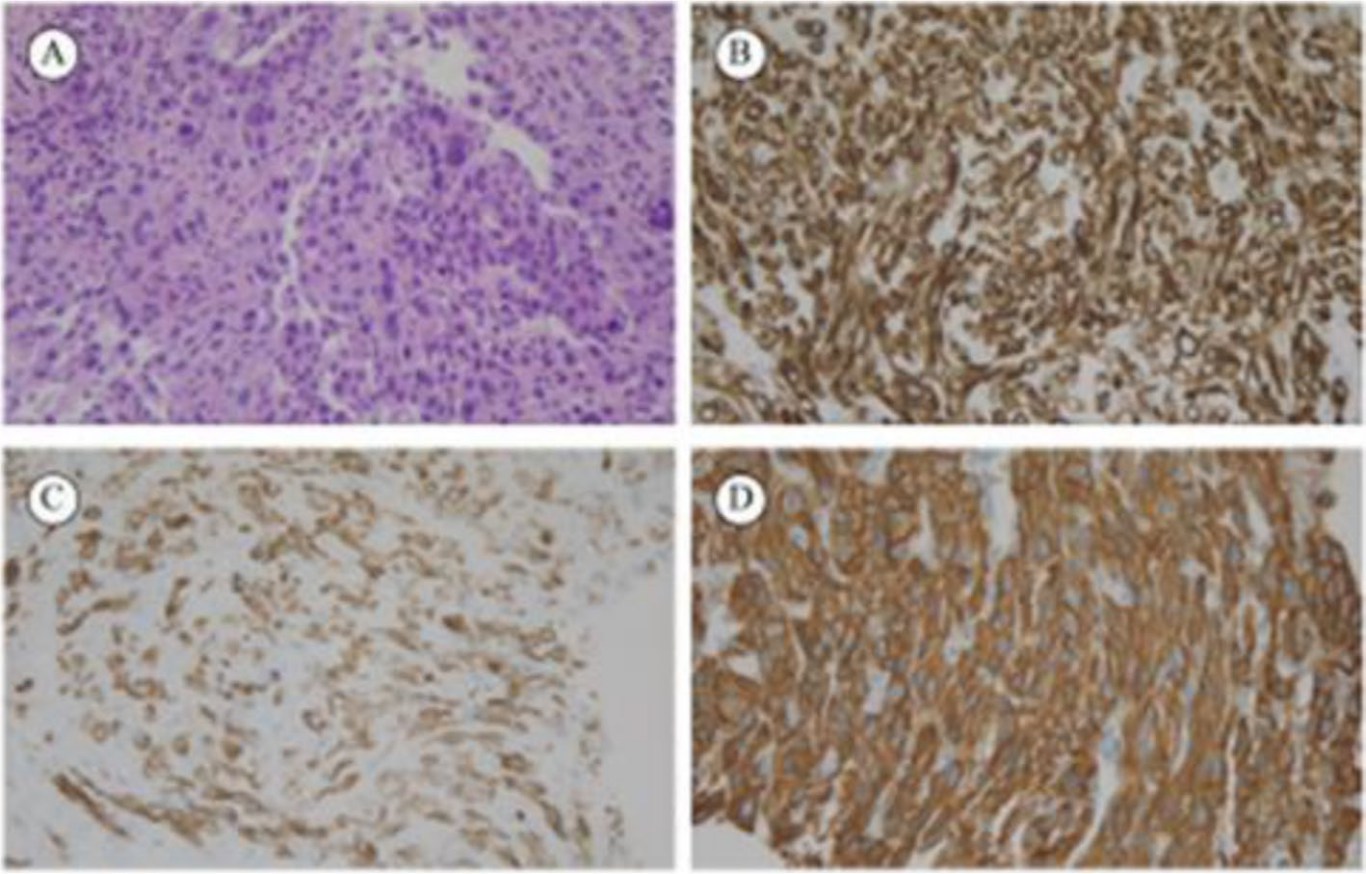
Pathological examination of the reported case. **A** Hematoxylin and eosin (200 ×). **B** CK7( +) (200 ×). **C** CK19 (partly +) (200 ×). **D** HER2 (3 +) (200 ×)

On 5 November 2019, a 64-year-old male Chinese patient was admitted to Peking Union Medical College Hospital for a liver tumor found by ultrasound during physical examination, without positive symptoms. The patient had a history of hepatitis B virus infection for 29 years. Magnetic resonance imaging (MRI) showed a 6-cm tumor in the right liver. The liver function test was normal. Among the serum tumor markers, cancer antigen 19–9 (CA19-9) was 103.9 U/ml (normal range was < 34 U/ml), carcinoembryonic antigen (CEA) was 7.1 ng/ml (normal range was < 5 ng/ml), and alpha-fetoprotein (AFP) was 12.5 ng/ml (normal range was < 20 ng/ml). The patient was not a candidate for surgical intervention because PET-CT showed bilobar disease with innumerable liver lesions and metastatic lymph nodes. After tumor biopsy, pathological diagnosis confirmed poorly differentiated adenocarcinoma, with positive immunohistochemical (IHC) staining for CK7( +), CK19 (partly +), HER2 (3 +), and MUC1( −) (Fig. 1). The status of PD-L1 was negative counted by combined positive score (CPS). Next-generation sequencing (NGS) verified the amplification of the HER2 gene, and the copy number variation of HER2 was 102. The tumor mutational burden was 3.6 Muts/Mb, and microsatellite instability was stable microsatellites tested by polymerase chain reaction. Finally, the patient was diagnosed with poorly differentiated ICC with multiple lymph node metastases (including in the hilar lymph nodes, posterior pancreatic head, para-aortic lymph nodes, and right cardiac-diaphragmatic angle) harboring HER2 amplification (cT2N1M0, stage 3b).

Platinum-based chemotherapy was recommended. Because the patient was worried about the adverse events of chemotherapy, he received pyrotinib (administered orally each day at a dose of 400 mg per day) and tegafur (administered orally each day at a dose of 40 mg per day). However, after 6 months of treatment, PET-CT showed enlargement of the primary lesions and metastatic lymph nodes, with the appearance of bone metastasis in the lumbar spine and pubis, and the disease was evaluated by the PERCIST to have progressed. The progression-free survival with first-line treatment was 6 months. The patient developed grade 3 adverse reactions, including skin rash, hypertension, and increased bilirubin concentration, but no grades 4 or 5 adverse reactions were observed.

**Fig. 2:**
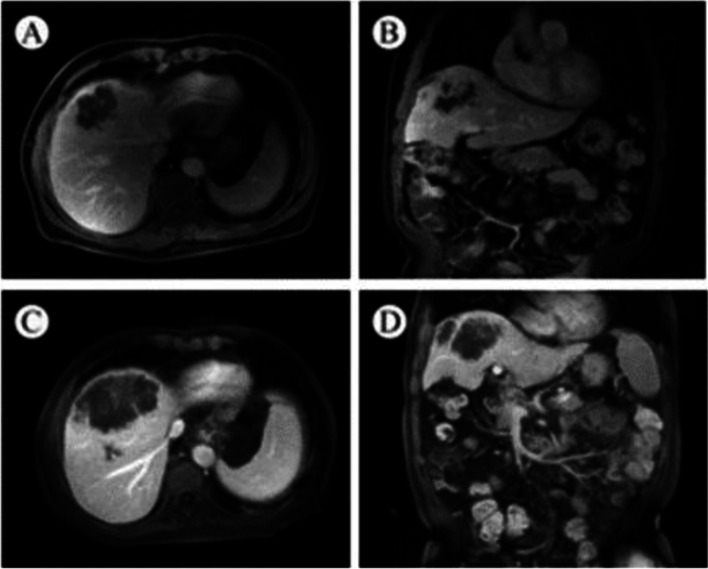
MRI showed pseudo-progression of the primary tumor. **A** and **B** MRI in January 2021. **C** and **D** MRI in July 2021. Abbreviations: MRI, magnetic resonance imaging

After a discussion with the patient and his family, second-line pyrotinib (administered orally each day at a dose of 80 mg per day) and lenvatinib (administered orally each day at a dose of 12 mg per day) in combination with pembrolizumab (administered 2 mg/kg as an intravenous infusion over 30 min every 3 weeks) were used. After 2 months, the lesions in the liver were found to have increased in size on MRI (Fig. 2), but the tumor marker levels decreased. The multidisciplinary team including the surgery, internal medicine oncology, and radiology departments reevaluated the efficacy of treatment. Pseudo-progression was suspected based on the performance improvement and decrease in tumor markers, so the patients continued with the treatment. Four months later, PET-CT showed that the primary lesions had shrunk, accompanied by CA19-9 and CEA reductions (CA19-9 levels decreased from 185 to 57.3 U/mL, the CEA level decreased from 8.1 to 2.6 ng/mL), and the efficacy was evaluated as a partial response (PR). In June 2020, lenvatinib was reduced to 8 mg per day because of increased bilirubin levels. The PET-CT evaluation in May 2021 revealed PR. However, the PET-CT on November 2021 showed that the patient developed subclavian lymph node metastasis. The progression-free survival with second-line treatment was 17 months. After using the three drugs, the patient developed grade 3 adverse reactions, including fatigue, irregular bowel movement, skin rash, hypertension, and increased bilirubin concentration, but no grades 4 or 5 adverse reactions were observed.

**Fig. 3:**
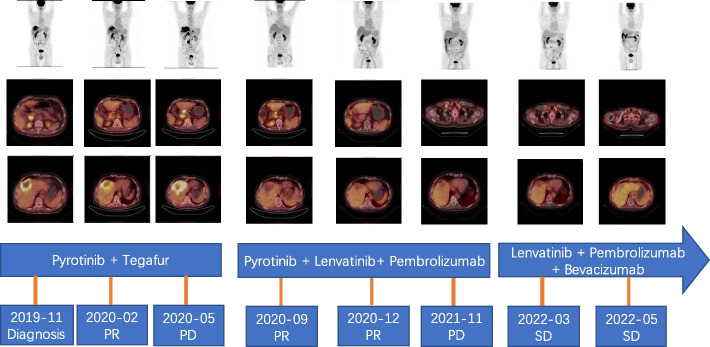
Images of the patient during treatment. Abbreviations: SD, stable disease; PD, progressive disease; PR, partial response

**Fig. 4:**
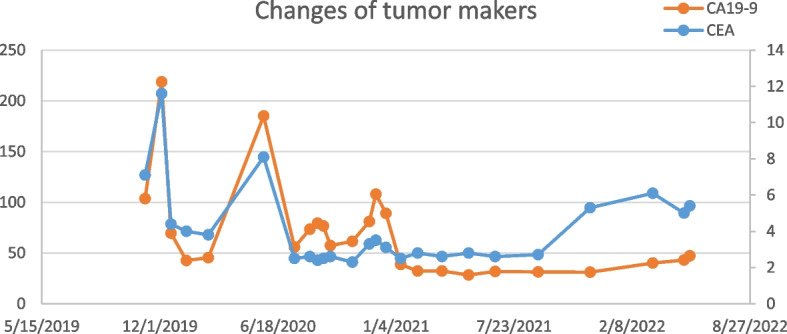
Preoperative and postoperative tumor marker results: carcinoembryonic antigen (CEA) and carbohydrate antigen 199 (CA19-9). Abbreviations: SD, stable disease; PD, progressive disease; PR, partial response

For the third line of therapy, lenvatinib (administered orally each day at a dose of 12 mg per day) and pembrolizumab (administered 2 mg/kg as an intravenous infusion over 30 min every 3 weeks) in combination with bevacizumab (15 mg/kg intravenously every 3 weeks) were used. The recent evaluation by PET-CT in May 2022 showed stable disease (SD). The progression-free survival with third-line treatment is 6 months thus far. After using the three drugs, the patient developed grade 3 adverse reactions, including fatigue, irregular bowel movement, skin rash, and proteinuria, but no 4 or 5 adverse reactions were observed. The changes of images and tumor makers during the treatemnt for this patient were summarized in Figs. 3 and 4. Written informed consent to publish the case details was obtained from the patient.

## Discussion

In the published literature, this is the first case report to show a clinical response in advanced BTC that progressed after first-line therapy. The combination of pyrotinib with pembrolizumab and lenvatinib identified here may represent a promising strategy for advanced BTC patients who have high levels of HER2. Our patient received second-line therapy without chemotherapy and achieved a clinical response. Currently, he has stable disease, and the patient has lived for approximately 29 months since he was diagnosed.

At present, gemcitabine combined with cisplatin is widely used as the first-line treatment in patients with advanced ICC [8]. However, the prognosis is very poor (median overall survival of 11.7 months and median progression-free survival of 8 months), and 70.7% of patients had grades 3 or 4 toxic effects [8]. Our patient worried about the adverse events of chemotherapy and received pyrotinib and tegafur as first-line treatment, and the progression-free survival with this first-line treatment was 6 months, with no serious adverse events. HER2-targeted therapy provides another choice for first-line therapy for patients with HER2 amplification, with a comparable progression-free survival and fewer adverse events.

Currently, there is no standard second-line treatment for advanced BTC. The ABC-06 clinical trial with FOLFOX chemotherapy reported a median progression-free time of 4.0 months and the median OS of 6.2 months [9]. HER2-targeting antibody has been shown to be as a new therapeutic option against advanced BTC recently [10]. Dual-HER2-targeted therapy for treating HER2-positive metastatic BTC with the median progression-free time was 4.0 months, and the median OS was 10.9 months [6]. Compared with chemotherapy, HER2-targeted therapy appeared as a better scheme for HER2-positive metastatic BTC. However, both results were significantly shorter than 17 months of our study. The continuation of HER2-targeted therapy showed a significant improvement in overall response and time to progression in patients who experienced progression during HER2-targeted therapy [11]. As a result, we continued to use the pyrotinib for the second-line treatment. Our patient achieved clinical response with combination therapy, indicating that our patient acquired an effective treatment. In conclusion, HER2-targeted therapy combined with pembrolizumab and lenvatinib was probably effective for HER2 amplification ICC.

Bevacizumab, a VEGF inhibitor, has been evaluated in advanced biliary tract cancers in combination with chemotherapy, which resulted in an overall response rate of 41% with a median PFS of 7.6 months and a median OS of 14.2 months in patients with ICC [12]. Moreover, bevacizumab was shown to have the effect enhancing the role of immunotherapy for hepatocellular carcinoma [13]. As a result, we added bevacizumab for third-line treatment. To date, the patient has had stable disease for approximately 6 months. Because the patients had a single metastasis of subclavian lymph node after the second-line treatment, we have planned radiotherapy for the lymph node as the next step in treatment.

Our previous reports have shown that pembrolizumab combined with lenvatinib showed a good response in advanced BTC, with an objective response rate (ORR) of 25% and a disease control rate (DCR) of 78.1% [14]. Recently, an article published in Nature showed that EGFR activation limits the response of liver cancer to lenvatinib in HCC [15]. HER2 is a member of the human epidermal growth factor receptor (ERBB or HER) family. The human epidermal growth factor receptor (ERBB or HER) family includes HER1 (also known as EGFR), HER2 (ERBB2), HER3, and HER4. The combination of the EGFR inhibitor gefitinib and lenvatinib displayed potent antiproliferative effects in HCC tumors [16]. The inhibition of fibroblast growth factor receptor (FGFR) by lenvatinib treatment leads to feedback activation of the EGFR–PAK2–ERK5 signaling axis in patients with high EGFR expression [15]. HER2 can also combine with EGFR and activate the EGFR–PAK2–ERK5 signaling axis. Thus, HER2 amplification could be another resistance mechanism to lenvatinib. The good response in our patient suggested that combination HER2-targeted therapy and lenvatinib was a potentially effective treatment for cholangiocarcinoma with HER2 amplification. The more specific mechanism needs further elucidation in clinical studies and basic studies.

Pseudo-progression is a unique response pattern to immune checkpoint inhibitors and includes an increase in the size of tumor lesions with tumor necrosis, prior to a subsequent decrease in tumor burden [17]. The presence of a response after an increase in total tumor burden and in the presence of new lesions was associated with favorable survival in pseudo-progression [18]. Although our patient had an increased tumor burden after using immunotherapy, he had improved performance and a decrease in tumor markers. As a result, he was suspected to have pseudo-progression after receiving immune checkpoint inhibitors. Pseudo-progression indicated a favorable response to immunotherapy in our patient. The good response of our patient to immunotherapy could be explained by the fact that the inhibition of angiogenesis from targeting VEGF/VEGFR could enhance the antitumor efficacy of immunotherapy [19, 20]. The relationship between HER2-targeted therapy and immunotherapy needs to be further explored in future studies.

In summary, adding pyrotinib to pembrolizumab and lenvatinib offered a chemotherapy-free option and could achieve complete tumor suppression with no serious adverse events for this patient with BTC with HER2 amplification.

## Data Availability

The data and materials used and/or analyzed during the current study are included in this article. The data are available from the corresponding author on reasonable request.
